# Physico-Chemical Properties and Performance of Functional Monomers Used in Contemporary Dental Adhesive Technology

**DOI:** 10.3290/j.jad.c_2297

**Published:** 2025-10-07

**Authors:** António H.S. Delgado, Mohammed H. Ahmed, Marta Nunes Ferreira, Ana Mano Azul, Mário Polido, Kumiko Yoshihara, Bart Van Meerbeek

**Affiliations:** a António H.S. Delgado Assistant Professor, Egas Moniz Center for Interdisciplinary Research (CiiEM), Egas Moniz School of Health & Science, Campus Universitário, Monte de Caparica, Almada 2829-511, Portugal; Division of Biomaterials and Tissue Engineering, UCL Eastman Dental Institute, UCL, London, UK. Study conception, design, data collection, drafting and reviewing the manuscript.; b Mohammed H. Ahmed Dentist, Tanta University, Faculty of Dentistry, Department of Dental Biomaterials, Tanta, Egypt. Study design, data collection, drafting and reviewing the manuscript.; c Marta Nunes Ferreira Dentist, Egas Moniz Center for Interdisciplinary Research (CiiEM), Egas Moniz School of Health & Science, Campus Universitário, Monte de Caparica, Almada 2829-511, Portugal. Data collection, drafting and reviewing the manuscript.; d Ana Mano Azul Associate Professor, Egas Moniz Center for Interdisciplinary Research (CiiEM), Egas Moniz School of Health & Science, Campus Universitário, Monte de Caparica, Almada 2829-511, Portugal. Study supervision, data curation, and reviewing the manuscript.; e Mário Polido Associate Professor, Egas Moniz Center for Interdisciplinary Research (CiiEM), Egas Moniz School of Health & Science, Campus Universitário, Monte de Caparica, Almada 2829-511, Portugal. Study supervision, data curation, and reviewing the manuscript.; f Kumiko Yoshihara Senior Researcher, National Institute of Advanced Industrial Science and Technology (AIST), Health and Medical Research Institute, Takamatsu, Kagawa, Japan; Okayama University, Graduate School of Medicine, Dentistry and Pharmaceutical Sciences, Department of Pathology & Experimental Medicine, Okayama, Japan. Study supervision, data curation, and reviewing the manuscript.; g Bart Van Meerbeek Full Professor, Department of Oral Health Sciences, KU Leuven, BIOMAT & UZ Leuven, Dentistry, Leuven, Belgium. Study design, data collection, drafting and reviewing the manuscript.

**Keywords:** 10-MDP, adhesion in dentistry, dental adhesion, etching, functional monomer, hybrid layer, monomer, self-adhesive, self-etching adhesive, self-etching system, universal adhesive

## Abstract

This comprehensive literature-based review critically examines the physico-chemical properties of functional monomers present in contemporary dental adhesive formulations or dental materials relying on self-adhesive technology. A qualitative synthesis of evidence was conducted through searches in PubMed, Scopus, and LILACS over a 20-year period (2005–2025), without language restrictions. Data on the chemical structure, composition, adhesive performance, pKa, etching efficacy, polymerization, mechanical properties, toxicity, and hydrolytic stability/degradation were analyzed from peer-reviewed studies and manufacturer technical information. Several relevant acidic functional monomers are covered, but key players include 10-MDP, GPDM, and 4-META. Notably, 10-MDP emerged as the most prevalent monomer in commercial adhesives, appearing in nearly 50% of the current adhesives in the market. Its superior adhesive performance and longevity stem from its unique chemical characteristics, whereas other commercial acidic monomers, including GPDM and 4-META, are still present in many adhesive formulations despite their structural limitations and comparatively lower bonding efficacy. Understanding the chemical composition of dental adhesives is essential for achieving improved clinical outcomes and driving material development. This knowledge allows clinicians to select adhesive materials based on performance requirements and informs future innovations to address challenges such as degradation pathways, biocompatibility, and their overall long-term bonding efficacy.

Research concerning dental biomaterials has attained significant advancement in the past decades, resulting in a maximization of clinical longevity, thereby extending the lifetime of restored teeth.^65^ The development of strong and durable adhesive strategies and formulations, to bond to both enamel and dentin simultaneously, is a noteworthy success factor in modern operative dentistry.^65,78,103^ Despite these technological advancements, there are many challenges in dental bonding that remain. Bond degradation is a particular concern. In dentin, it is possible to consider two major degradation pathways: (1) water sorption of adhesives that may lead to hydrolysis of chemical groups susceptible to hydrolytic degradation and subsequent elution; (2) interfacial enzymatic biodegradation induced by matrix metalloproteinases (MMPs) and cysteine cathepsins and/or bacterial biofilm by-products. All of these may lead to consequent bond deterioration.^95,96^ Successful dental restorations depend not only on these factors but also on often-overlooked details such as technique sensitivity during material placement, the operator’s experience, and the chemical composition of the materials, all of which can limit the bonding performance of adhesives. Therefore, comprehensive knowledge of adhesives and adherence to use recommendations are crucial to ensure proper placement.

Dental adhesives are a solvated heterogeneous monomeric blend of hydrophobic and hydrophilic ingredients that may contain a suspension of filler particles.^71^ Typically, the mixture is composed of crosslinking dimethacrylate monomers, such as bisphenol-A glycidyl dimethacrylate (Bis-GMA) and triethylene glycol dimethacrylate (TEGDMA) as a viscosity regulator, but also co-solvent monomethacrylate monomers such as 2-hydroxyethyl methacrylate (HEMA),^5,^ in addition to the most commonly used camphorquinone/amine photo-initiator system. However, to achieve chemical adhesion, manufacturers also include monomers capable of chemical interaction with the tooth substrate, named acidic functional monomers.^65^ Examples of these are 4-methacryloxyethyl trimellitic acid (4-META), 10-methacryloxydecyl dihydrogen phosphate (10-MDP), or glycerophosphate dimethacrylate (GPDM), being the most common functional monomers contained in contemporary adhesives. These functional monomers are characterized by at least one polymerizable methacrylate group and an acidic functional group (eg, carboxylic or phosphate group in the examples above), which can serve different purposes. Thanks to these functional monomers, surface wetting, (partial) etching, and chemical interaction with the tooth substrate become possible.^116,128,132^ Differences between functional monomers and their performance are generally explained by their differential chemical structure.^56,120^

Over the years, different formulations gave rise to distinct dental adhesive generations that can be applied in either one of two classical modes: etch-and-rinse (E&R) and self-etch (SE), or offering the optional applicability of both of them, as is the case for the newest generation of universal adhesives (UAs).^1^ Effective tooth bonding fundamentally requires adequate surface wetting and micromechanical retention, ideally complemented by chemical binding (Fig 1).

**Fig 1:**
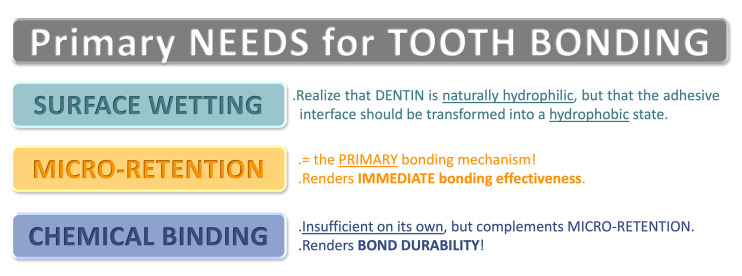
Primary needs for tooth bonding.

The E&R bonding mode consists of about 15-s phosphoric-acid application followed by a thorough water-rinse phase. Next, a monomer mixture in one or two separate steps is applied, constituting a two-step or three-step E&R adhesive, respectively.^81^ This process allows resin to diffuse/infiltrate within the etched/micro-retentive sites, creating a strong micromechanical interlocking mechanism, on which E&R adhesives heavily rely, a mechanism that is able to secure the most durable bond to enamel. This E&R approach results in a several micrometers deep hydroxyapatite (HAp)-depleted hybrid layer at dentin. While secondary chemical binding, such as van der Waals forces and hydrogen binding, will be involved, primary chemical binding to the exposed collagen is very unlikely. This collagen-rich E&R hybrid layer has been documented to be highly sensitive to time-dependent hydrolytic degradation. This is primarily related to incomplete saturation of the exposed collagen network with resin,^72^ and possibly also because of the absence of primary chemical interaction between the functional monomers and collagen.

The SE bonding mode, on the contrary, does not involve phosphoric-acid etching or water rinsing. It employs application of a hydrophilic-natured 1- or 2-step adhesive that relies primarily on a contained acidic functional monomer that is able to mildly (self-)etch and chemically bind to the mineral phase in dentin.^55,66^ Despite the resultant primary chemical binding benefit, SE bonding suffers some limitations: (1) potential interference of surface smear resulting from cavity preparation by bur; (2) high hydrophilicity that promotes water uptake at the adhesive interface with dentin, in particular for thin-film 1-step SE adhesives; and (3) insufficient enamel-bonding performance due to the lower acidity of SE adhesives, a convincing reason for practitioners to combine a selective enamel E&R mode with an SE bonding mode, being applied to etched enamel and (un-etched) bur-cut dentin.^55,66^

Bond durability plays an important role in adhesive dentistry, determining the success of the restorative procedure, which, at dentin, is reached by the formation of a stable, cohesive, and well-infiltrated three-dimensional hybrid layer (HL).^96,^ ^97^ The primary mechanism of dental adhesion still involves micromechanical diffusion interlocking at both enamel and dentin (Fig 1). In the latter, this is guaranteed by in-situ (co-)polymerization of resin monomers within a fully (E&R) or partially (SE) demineralized collagen network.^61,88^ Secondary mechanisms imply the creation of stable primary chemical binding, like ionic binding to minerals via dedicated functional monomers.^126,132^ Although chemical adhesion will not translate into high(er) bond strengths, such a mechanism is able to contribute to long-term stability of the adhesive interface as a complement to micromechanical retention (Fig 1), in particular when undertaken in HAp-rich SE hybrid layers. Contemporary UAs typically contain 10-MDP as one of the most effective functional monomers available. They thus enable chemical binding when used in SE bonding mode. Unlike traditional E&R adhesives that lack 10-MDP, UAs applied in E&R mode may also achieve chemical interaction at the base of the E&R hybrid layer, where residual HAp remains available. While chemical interaction between 10-MDP and collagen has been hypothesized in E&R hybrid layers, definitive experimental confirmation is still lacking. Overall, it is important to know that the long-term performance of current dental adhesive generations is material/product dependent. This requires the clinician to plan which adhesive to use and how, in accordance with knowledge of its chemical composition.

The chemical binding mechanism was clarified with the adhesion-decalcification (AD) concept, introduced by Yoshida et al (2001)^117^ (Fig 2). Functional monomers were found to either bond (“adhesion route”) to or decalcify (“decalcification route”) the tooth substrate. The chemistry of the monomers will dictate whether it is one or the other route. Depending on the diffusion rate of the formed calcium-acid complexes into solution, the acid will either remain attached to the HAp surface, with only limited decalcification involved, following the “adhesion route,” or the calcium-acid complex will de-bond, resulting in a substantial decalcification effect following the “decalcification route.” As a result, the less soluble calcium salt will provide the most intense and stable molecular bond to a HAp-based substrate ^41,117,120,132^

**Fig 2:**
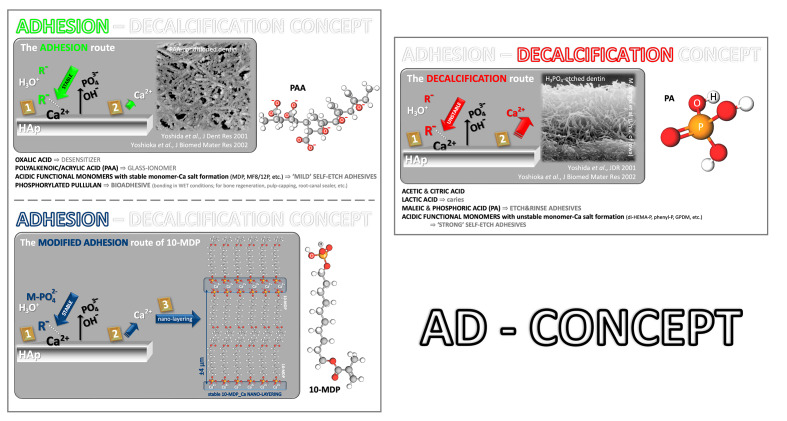
The adhesion-decalcification (AD) concept that dictates the interaction of functional mono/polymers with mineralized hard tissues.

Functional monomers can be found in SE adhesives and UAs, in some E&R formulations, in self-adhesive composite cements, but also, most recently, in self-adhesive restorative composites.^55,65,71^ Over the years, dental adhesives have been improved and simplified, in order to achieve better adhesion mechanisms, while largely becoming time-efficient for clinicians and patients.^22^ The incorporation of functional monomers allows adhesives to have an extra bond strategy through chemical adhesion. Their composition will influence certain bonding determinants such as hydrophobicity/hydrophilicity, pH, thickness/quality of the hybrid layer, chemical adhesion stability, but also polymerization kinetics and mechanical properties.^23,57,58,109^ In fact, there are several studies that evaluate adhesive performance, demineralization efficacy, pH, and stability of selected functional monomers.^26,44,74,108,120^ Also, new functional monomers and materials are currently under development.^21,136^ Still, there are no updated reviews that gather and map important chemical information concerning these monomers, nor do they compare their principal characteristics. It is therefore important to gather such data to better understand and compare differences in performance seen with adhesive materials, while also contributing to the future development of dental adhesive technology.

## METHODS

### Information Sources

To identify relevant papers with data concerning functional monomers used in current adhesive formulations, a comprehensive review was performed, from the period of July 2024 to March 2025, using the following databases: PubMed/Medline, Scopus, and LILACS. The search strategy mainly focused on full papers published in the last 20 years (2005–2025), as contemporary materials are the aim of this qualitative synthesis, without any language restrictions, although older reference papers were also retrieved. This review does not follow a systematic or scoping approach as it does not answer an interventive question but rather serves to map and gather information concerning adhesive monomers used in material formulations. Full-text papers containing data such as adhesive performance, etching efficacy, interaction mechanisms of monomers to substrates, polymerization, toxicity, and degradation experiments were included, as these enable relevant comparisons.

To identify functional monomers present in contemporary adhesives, information sheets and safety datasheets provided by the respective manufacturers, belonging to dental adhesives commercialized by major dental material companies, were examined. These include, but are not limited to 3M Oral Care (Seefeld, Germany; St. Paul, MN, USA; now Solventum), Bisco (Schaumburg, IL, USA), Coltène-Whaledent (Altstätten, Switzerland), Dentsply Sirona (Konstanz, Germany), DMG (Hamburg, Germany), GC (Tokyo, Japan), Heraeus Kulzer (Hanau, Germany), Ivoclar (Schaan, Liechtenstein), Kerr (Orange, CA, USA), Kuraray Noritake (Tokyo, Japan), Shofu (Kyoto, Japan), SDI (Bayswater, Victoria, Australia), Tokuyama (Tokyo, Japan), and Voco (Cuxhaven, Germany).

### Chemical Parameters

The software ChemBioDraw Ultra 19.1 for Mac (Perkin-Elmer, Waltham, MA, USA) was used to determine the chemical features of the functional monomers identified earlier. This software enables users to draw the chemical structure of each monomer and to estimate the following parameters: molecular weight (g/mol), partition coefficient (LogP), and aqueous solubility (LogS; mol/L). The molecular weight impacts monomer reactivity, polymerization kinetics/shrinkage,^79^ and adhesion diffusion capability.^85^ The partition coefficient is relevant for indicating hydrophobicity/hydrophilicity, which significantly affects phase separation, diffusion within tooth substrates, and adhesion stability under moisture challenge.^80^ Furthermore, the software provides an estimation of aqueous solubility, a parameter relevant for predicting the potential of monomers to dissolve or leach into aqueous environments, a phenomenon that could impact material stability and indirectly influence biological interactions.^25^

### Qualitative Synthesis

#### Comparison of adhesives

Table 1 presents adhesive materials containing functional monomers, based on technical information provided by manufacturers. Of the 71 adhesives identified, 11 did not disclose at least one functional monomer, citing it as a trade secret. Among the adhesives analyzed, 10-MDP was the most frequently reported functional monomer, found in ~44% (31 out of 71) of the adhesives identified in the literature.

**Table 1:** Composition of commercial dental adhesives identified from technical information provided by the respective dental manufacturers, as being used in contemporary practice and listed in alphabetical order, per adhesive class

Adhesive	Manufacturer	Application steps	Contained functional monomer
**ETCH & RINSE ADHESIVES (E&R)**
Scotchbond 1 XT	3M Oral Care/Solventum (St. Paul, MN, USA)	2-step	polyalkenoic acid co-polymer
Adper Scotchbond Multi-Purpose	3M Oral Care/Solventum	3-step	polyalkenoic acid co-polymer
All-Bond 3	Bisco (Schaumburg, IL, USA)	3-step	PENTA
Clearfil Photo Bond	Kuraray Noritake (Tokyo, Japan)	3-step	10-MDP
Ecosite Bond	DMG (Hamburg, Germany)	2-step	10-MDP
ExciTE F DSC	Ivoclar (Schaan, Liechtenstein)	2-step	phosphonic acid acrylate
Gluma Bond 5	Heraeus Kulzer (Hanau, Germany)	2-step	4-META
iBond Total Etch	Heraeus Kulzer	2-step	4-META
One-Step	Bisco	2-step	BPDM
Optibond FL	Kerr (Orange, CA, USA)	3-step	GPDM, MMEP
Optibond Solo Plus	Kerr	2-step	GPDM
Prime & Bond NT	Dentsply Sirona (Konstanz, Germany)	2-step	PENTA
ProBOND Total-etch	Dentsply Sirona	2-step	PENTA
Solobond M	Voco (Cuxhaven, Germany)	2-step	acidic adhesive monomer
Solobond Plus	Voco	3-step	HPMA
Tetric N-Bond	Ivoclar	2-step	10-MDP
Tokuyama EE Bond	Tokuyama (Tokyo, Japan)	2-step	HEMA-P
**SELF-ETCH ADHESIVES (SE)**
Easy Bond	3M Oral Care/Solventum	1-step	6-MHP
Prompt L-Pop	3M Oral Care/Solventum	1-step	HEMA phosphates
Beautibond	Shofu	1-step	phosphonic & carboxylic acid monomers
Clearfil Liner Bond 2V	Kuraray Noritake (Tokyo, Japan)	2-step	10-MDP
Clearfil S3 Bond Plus	Kuraray Noritake	1-step	10-MDP
Clearfil SE Bond 2	Kuraray Noritake	2-step	10-MDP
Clearfil SE Protect Bond	Kuraray Noritake	2-step	10-MDP, MDPB
Contax	DMG	2-step	unknown
FL-Bond II	Shofu (Kyoto, Japan)	2-step	4-AETA, phosphonic acid monomer
Futurabond DC	Voco	1-step	acidic adhesive monomer
Futurabond M	Voco	1-step	acidic adhesive monomer
Futurabond NR	Voco	1-step	acidic adhesive monomer
G-aenial Bond	GC	1-step	phosphoric acid ester monomer
G-Bond	GC	1-step	4-MET, 10-MDP
H-Etchbond	Heydent (Kaufering, Germany)	1-step	10-MDP, 4-META
iBond Self Etch	Heraeus Kulzer	1-step	4-META
Maxbond LC	J Morita (Osaka, Japan)	1-step	phosphate esters
One-Up Bond F Plus	Tokuyama	1-step	MAC-10
Optibond All-In-One	Kerr	1-step	GPDM
Tokuyama Bond Force II	Tokuyama	1-step	HEMA-P
Xeno III	Dentsply Sirona	1-step	Pyro-EMA, PEM-F
Xeno IV One	Dentsply Sirona	1-step	PENTA
**UNIVERSAL ADHESIVES (UA)**			
AdheSE Universal	Ivoclar	2-/1-/2-step	10-MDP, methacrylated carboxylic acid polymer
All-Bond Universal	Bisco	2-/1-/2-step	10-MDP
Ambar Universal	FGM (Joinville, Brazil)	2-/1-/2-step	10-MDP
BeautiBond Xtreme	Shofu	2-/1-/2-step	4-AETA, phosphonic acid monomer
Clearfil Universal Bond Quick	Kuraray Noritake	2-/1-/2-step	10-MDP
Clearfil Universal Bond Quick 2	Kuraray Noritake	2-/1-/2-step	10-MDP
Ecosite Bond	DMG	2-/1-/2-step	10-MDP
Futurabond M+	Voco	2-/1-/2-step	acidic adhesive monomer
Futurabond U	Voco	2-/1-/2-step	acidic adhesive monomer
G2 Bond Universal	GC	2-/1-/2-step	4-MET, 10-MDP, MDTP
G-Premio Bond	GC	2-/1-/2-step	4-MET, 10-MDP, MDTP
GC Solare Universal Bond	GC	2-/1-/2-step	phosphoric acid ester monomer
Gluma Bond Universal	Heraeus Kulzer	2-/1-/2-step	10-MDP, 4-META
Healbond Max	Elsodent (Herblay, France)	2-/1-/2-step	10-MDP
Healbond MP	Elsodent	2-/1-/2-step	10-MDP
Ipera Bond Universal	Itena Clincal (Villepinte, France)	2-/1-/2-step	10-MDP, 4-META
iBond Universal	Heraeus Kulzer	2-/1-/2-step	4-META
K-Bond + Universal	Kiyomi (Gijón, Spain)	2-/1-/2-step	10-MDP
Luxabond Universal	DMG	2-/1-/2-step	10-MDP
Nova Compo-B Plus	Imicryl Dental (Konya, Turkey)	2-/1-/2-step	10-MDP, 4-META
One-Coat 7 Universal	Coltène-Whaledent (Altstätten, Switzerland)	2-/1-/2-step	10-MDP
Optibond eXTRa Universal	Kerr	2-/1-/2-step	GPDM
Optibond Universal	Kerr	2-/1-/2-step	GPDM
Optibond Universal 360	Kerr	2-/1-/2-step	GPDM, 10-MDP
Palfique Universal Bond	Tokuyama	2-/1-/2-step	phosphate acid ester monomer
Peak Universal Bond	Ultradent (South Jordan, UT, USA)	2-/1-/2-step	Dymetech™ phosphate monomer
Prime & Bond Active	Dentsply Sirona	2-/1-/2-step	PENTA
Prelude One	Zest Dental Solutions (Carlsbad, CA, USA)	2-/1-/2-step	Methacrylate phosphate monomer
ProLink Universal	Silmet (Yehuda, Israel)	2-/1-/2-step	10-MDP, 4-MET
Scotchbond Universal	3M Oral Care/Solventum	2-/1-/2-step	10-MDP, polyalkenoic acid polymer
Scotchbond Universal Plus	3M Oral Care/Solventum	2-/1-/2-step	10-MDP, polyalkenoic acid polymer
Tokuyama Universal Bond II	Tokuyama	2-/1-/2-step	HEMA-P, 10-MDP
Zipbond	SDI (Bayswater, Australia)	2-/1-/2-step	10-MDP

Abbreviation in alphabetical order: 4-AETA: 4-acryloyloxethyltrimellitic anhydride; 4-META/4-MET: 4-methacryloxyethyl trimellitic anhydride/4-methacryloxyethyl trimellitic acid; 6-MHP: 6-methacryloyloxyhexyl dihydrogen phosphate; BPDM: biphenyl-dimethacrylate; GPDM: glycerol-phosphate dimethacrylate; HEMA-P: 2-hydroxyethyl methacrylates phosphate; HPMA: 2-hydroxypropyl Methacrylate; MAC-10: 11-methacryloyloxy-1,10-undecanedicarboxylic acid; MDTP: 10-methacryloyloxydecyl dihydrogen thiophosphate; MDP/10-MDP: 10-methacryloyloxydecyl dihydrogen phosphate; MDPB: methacryloyloxydodecylpyridinium bromide; MMEP: mono-2-methacryloyloxyethyl phthalate; PEM-F: pentamethacryloyloxyethylcyclohexaphosphazene monofluoride; PENTA: dipentaerythritol penta-acrylate phosphate; Phenyl-P: 2-methacryloyloxyethyl phenyl phosphoric acid; Pyro-EMA: tetramethacryloyloxyethyl pyrophosphate.

#### Chemical properties of existing functional adhesive monomers

**Fig 3:**
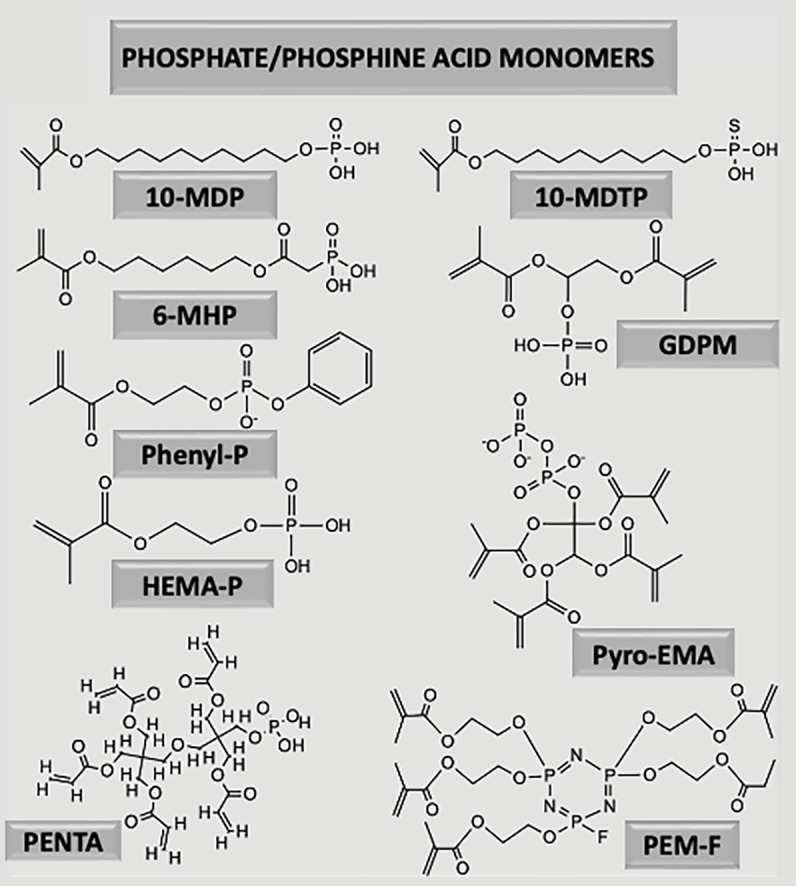

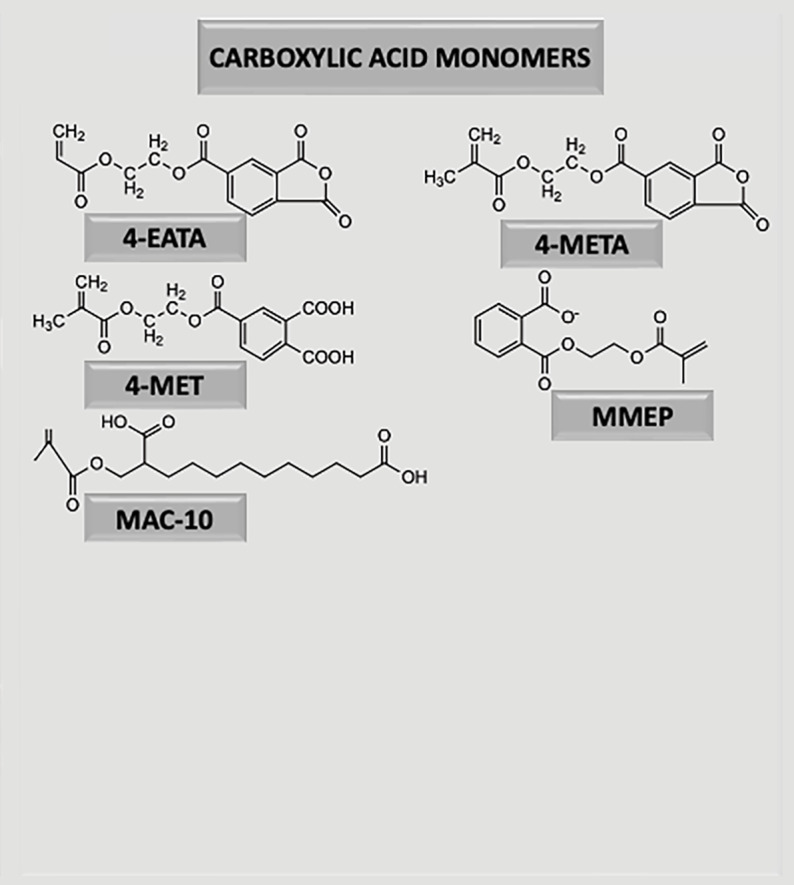
Chemical structure of the various functional monomers included in contemporary dental adhesives that were identified in this review.

A comparison between the chemical properties and structure of the most relevant functional monomers identified in the commercial adhesives present in Table 1, are given in Table 2 and Figure 3, respectively.

**Table 2:** Functional monomers and their respective chemical properties in terms of molecular weight (g/mol), partition coefficient (Log P), and aqueous solubility coefficient (Log S; mol/L)

Category	Functional monomer	Molecular weight (g/mol)	Log P^1^	Log S (mol/l)
Carboxylic acid monomers	phthalic acid monomethacrylate (MMEP)	277.3	0.47	–2.26
4-acryloyloxethyltrimellitic anhydride (4-AETA)	290.2	1.65	–2.90
4-methacryloxyethyl trimellitic acid (4-MET)^2^	322.3	1.71	–2.74
4-methacryloxyethyl trimellitic anhydride (4-META)	304.2	2.00	–3.04
Phosphate/phosphonic acid monomers	2-hydroxyethyl methacrylate phosphate (HEMA-P)	228.1	1.21	0.08
10-methacryloyloxydecyl dihydrogen thiophosphate (10-MDTP)	338.4	4.82	–3.84
pentamethacryloyloxyethylcyclohexaphosphazene monofluoride (PEM-F)	787.6	–	–4.86
tetramethacryloyloxyethyl pyrophosphate (Pyro-EMA)	539.3	–	–4.68
6-methacryloyloxyhexyl phosphonoacetate (6-MHP)	308.1	1.33	–1.49
dipentaerythritol penta-acrylate phosphate (PENTA)	604.5	2.60	–2.63
10-methacryloyloxydecyl dihydrogen phosphate (10-MDP)	322.3	4.09	–2.65
2-methacryloyloxyethyl phenyl phosphoric acid (Phenyl-P)	286.2	0.57	–2.20
glycerol-phosphate dimethacrylate (GPDM)	294.2	2.26	–0.79
^1^ LogP is unitless, ^2^ 4-MET forms upon reaction of 4-META in aqueous solutions.

The molecular weight of modern functional monomers ranges from 228 g/mol (2-hydroxyethyl methacrylate phosphate, HEMA-P) to 788 g/mol (pentamethacryloyloxyethylcyclohexaphosphazene monofluoride, PEM-F). The most hydrophilic monomer identified was mono-2-methacryloyloxyethyl phthalate (MMEP), followed by phenyl-P, whereas the most hydrophobic monomer was 10-methacryloyloxydecyl dihydrogen thiophosphate (10-MDTP), followed by 10-MDP. In terms of water solubility, PEM-F was the least soluble monomer, while HEMA-P exhibited the highest solubility.

#### Adhesive performance, hydrolytic stability, and longevity

**Fig 4:**
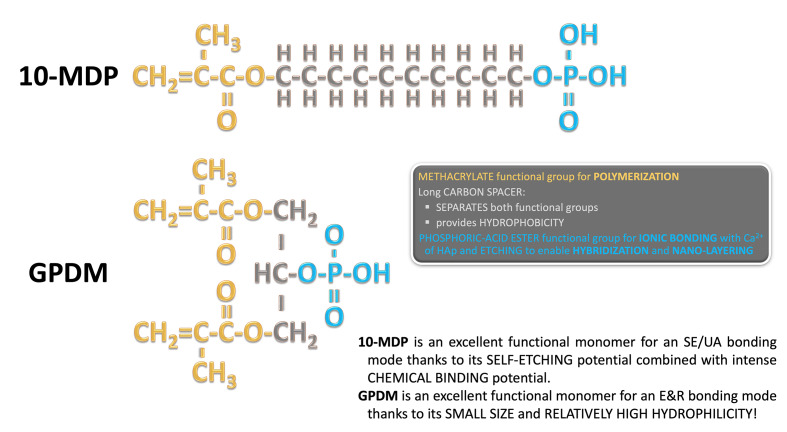
Molecular structure and basic structural features of the functional monomers 10-MDP and GPDM for self-etch (SE) and etch&rinse (E&R) bonding.

Functional monomers were initially developed and included in adhesives to improve their bonding mechanisms, by means of chemical adhesion, to enamel and dentin. Irrespective of the bonding mode employed, these functional monomers work following an SE strategy. Their quality, purity, and chemical nature will largely contribute to the bonding performance of the adhesive. In Figure 1, monomers are shown with their acidic functional groups, able to ionically interact with HAp. Commonly used monomers such as 10-MDP, GPDM, and phenyl-P possess a phosphate group as a chemically active functional group (Fig 4), while 4-MET (4-META) presents with two carboxylic acid groups. Activation of these acidic groups through ionization is required; thus, the presence of water in adhesive formulations is crucial for such acidic monomers to ionize and exert their function.^44^

The type of chemical moiety, its location in the molecule and subsequent steric hindrance, the size of the spacer chain, and the hydrophobicity/hydrophilicity balance have been proven to greatly influence the chemical bond obtained to dental substrates and its stability.^15,30,56,76^

Even though Kuraray Noritake first started to use the term “acidic adhesive monomer” with phenyl-P in 1976^53^, the functional monomer GPDM must have been one of the first functional monomers described in literature (Fig 4). In the mid-1950s, Buonocore et al (1956) reported on the bonding ability of GPDM, as contained in a pioneering sealing agent.^9^ Phenyl-P was later replaced by 10-MDP in adhesive formulations, as researchers found that the phosphate group at one molecular end, separated by a hydrophobic 10-CH2 carbon-spacer chain from the polymerizing methacrylate group at the other molecule end, facilitated and promoted long-lasting interactions with HAp (Fig 4). Phenyl-P was concurrently proven to have a lower chemical bonding efficacy compared to 4-META and 10-MDP. Furthermore, the aqueous solubility of the phenyl-P_Ca salt was found to be high and much more soluble than that of 4-META and in particular 10-MDP.^112^ Hence, phenyl-P lost its initial popularity and was no longer contained in newer adhesives. Monomers, such as GPDM, possess a substantially shorter spacer chain, bringing the phosphate and methacrylate active ends closer to each other, are smaller in size, and intrinsically more hydrophilic (Fig 4). Thanks to its small size (low molecular weight) and relatively high hydrophilicity, GPDM is an efficient functional monomer for an E&R bonding mode (Fig 4). GPDM was attributed lower bonding efficacy and a tendency to etch (demineralize) rather than adhere to mineralized substrates.^108^ Later, the nature of the resulting chemical bond was proven to determine its stability over time.^117,125,132^ Recent meta-analytical data of studies grouping 64 different dental adhesives has shown that 10-MDP has superior bonding performance when compared to 4-META, 4-MET, 6-MHP, penta-acrylate (PENTA), and pyro-EMA (P <0.01), while showing similar performance to PEM-F, 4-AET and MAC-10 or other acrylamide, polyacrylic or phosphonic acid derivatives, although it is important to mention that there is still insufficient data available, regarding these monomers, to actually prove similarities to 10-MDP in adhesive performance, long-term and clinically.^29^

**Fig 5:**
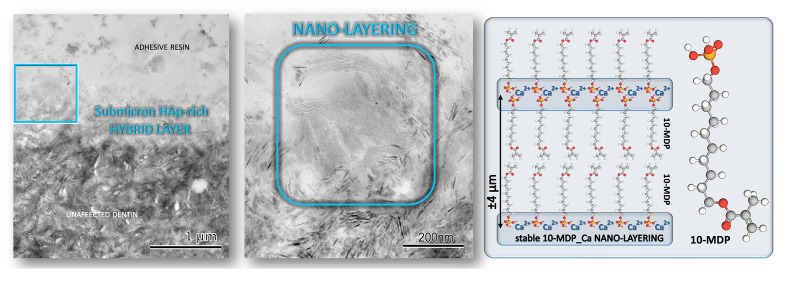
TEM photomicrographs showing the interfacial ultrastructure of a 10-MDP SE adhesive bonded to dentin, along with a schematic representation of the molecular structure of 10-MDP nano-layering.

In fact, it is today known that the typical long-chain monomer 10-MDP enables different 1.9-nm-sized 10-MDP molecules to self-align parallel to each other, hereby self-assembling in 3D in so-termed nano-layers (Figs 2, 4, and 5). Each nano-layer has a width of about 4 nm and consists of two rows of oppositely directed 10-MDP molecules with their polymerizing methacrylate group directed toward the middle of a nano-layer and their phosphate group to the nano-layer outside. One nano-layer is linked with an adjacent nano-layer through Ca, bridging two adjacent phosphate groups of two 10-MDP molecules belonging to two neighboring nano-layers. Self-evidently, nano-layers are strong and stable structures that add strength to the adhesive interface, as proven by atomic-force microscopy.^123^ In this way, nano-layering can contribute to the durability of the bond generated by 10-MDP-based SE adhesives to dentin.^1,65,123^ Alternatively, nano-layers formed within the adhesive resin adjacent to the hybrid layer could also be regarded as self-developed filler, a process triggered by Ca released from dentin through 10-MDP’s (self-)etching capacity.

Regarding hydrolytic stability, 10-MDP has also been documented to outperform its competitors, namely 4-META, GPDM, and phenyl-P. This has been proven in studies that tested immediate bonding of the monomers to HAp, rinsing, and ultrasonic cleaning,^118^ but also in studies featuring commercial adhesives containing these monomers, with micro-tensile bond strength tests conducted before/after significant thermocycling aging (30 k, 50 k, and 100 k cycles).^48^ The chemical structure of the monomers is paramount in determining their functional ability. In this respect, the impact of the length of the spacer chain (number of carbons) on the formation of monomer-calcium salts was investigated by Feitosa et al (2014), also in relation to the resulting hydrophilicity of the acidic monomers. Shorter spacer chains were associated with less hydrolytically stable and weaker monomer-calcium interactions. The presence of ester or ether groups in the monomers also affected hydrophilicity, which regulates bond longevity.^30^ Impurities in the monomer batch, or products of hydrolysis or time-dependent degradation of acidic monomers, were found to affect the formation of monomer-calcium salts. Achieving high purities in the synthesis of functional monomers is thus required to attain bond longevity.^124^ In regard to MMP activity, recently 10-MDP has been shown to inhibit MMPs at the interface,^91^ specifically inhibiting MMP-9 activity, reducing hydroxyproline (HYP) release, and minimizing nano-leakage, hereby further contributing to improved dentin-bonding durability.^50,134^ As found by Jin et al (2022), MMP-9 inhibition assays showed 10-MDP reduced enzyme activity by ~19%, while 10-MDP_Ca salt achieved ~49% inhibition. HYP release assays showed lower levels in both 10-MDP and 10-MDP_Ca groups, indicating less collagen breakdown. 10-MDP_Ca was more effective than 10-MDP alone, giving evidence of a synergistic protective effect.^50^

Table 3 summarizes studies found in the literature that varied the concentration of different functional monomers in experimental materials used to bond to different clinically relevant substrates.

**Table 3:** Summary of studies found in the literature that varied the concentration of different functional monomers in experimental primers/adhesives/cements/sealants/self-adhesive composites and tested adhesion, in different set-ups, to different substrates (enamel, dentin, zirconia, composite)

	Study	Purpose	Experimental groups	Result summary	Conclusion
Enamel	Wang et al (2013)^110^	To investigate the effect of priming time and 10-MDP concentration on micro-tensile bond strength to bovine enamel using a novel prime-and-rinse approach.	Experimental primers were formulated containing 10-MDP at 0.5, 1, 5, 10 and 20 wt%, and two neutral primers containing 5 wt% 10-MDP_Na or EDTA_2Na. Different priming times (5, 30, or 60 s) were also tested.	There was a significant influence of primer concentration and priming time when 10-MDP-containing primers were used, revealing an optimal priming time of 30 s and the best primer containing 20% 10-MDP.	The neutral primer (5% 10-MDP_Na) produced moderate bond strength, while 5% EDTA_2Na failed, suggesting that chemical bonding of 10-MDP to enamel hydroxyapatite crystallites could improve bond strength onto smooth enamel surfaces.
Hiraba et al (2023)^43^	To evaluate the effect of 10-MDP in MMA monomers on the bonding performance of TBB-initiated adhesive resins to human enamel for fixed prosthodontic applications.	Three experimental TBB-initiated self-polymerizing resins containing 10-MDP in different concentrations (i.e., 10-MDP/MMA-TBB resins) were used as luting agents (1.0, 1.7 and 2.0 mol% 10-MDP).	10-MDP/MMA-TBB resins demonstrated significantly greater bond durability to enamel compared to other resins, with or without etching. Thermocycling results indicated that 1.7 and 2.0 mol% 10-MDP concentrations were optimal for non-etched specimens, both before and after aging.	The optimal 10-MDP concentrations in the 10-MDP/MMA solution were suggested to be 1.7 and 2.0 mol% for adhesion of TBB polymerization-initiated resin to human enamel.
Yun et al (2025)^135^	To produce sealants containing mesoporous bioactive glass (MBG) and 10-MDP, and compare them on enamel remineralization and adhesion.	A solution containing either 5 wt% MBG, 5 wt% 10-MDP, or both, was incorporated into experimental resin-based sealants. Adhesive properties (shear bond strength: SBS) and remineralization potential (microhardness, XRD, FE-SEM) were evaluated.	10-MDP significantly improved SBS, while MBG promoted enamel remineralization. The combined MBG/10-MDP group showed synergistic effects: improved microhardness, crystal deposition, and better adhesion. However, 10-MDP alone reduced flexural strength.	Although 10-MDP improves SBS, it may compromise flexural strength. An optimal balance of MBG and 10-MDP is crucial for achieving enhanced remineralization and adhesion.
Dentin	Hasegawa et al (1989)^40^	To investigate the contraction gap in dentin by treating it with different concentrations of functional monomers in experimental primers.	A solution containing either one of four acidic monomers, mono- methacryloxyethyl succinate (MES), dimethacryloxyethyl phosphate (DMEP), tertiary butylacrylamide sulfonic acid (‘rBAS), and 4-META, at concentrations of 0.1, 0.2 or 0.3M diluted in 35% HEMA was prepared.	More than half of the specimens pre-treated with the experimental primers showed continuous marginal adaptation, comparable to the control.	These four monomers showed comparable results, being effective as self-etching primers.
Watanabe et al (1994)^111^	To demonstrate, by testing tensile bond strength and TEM, the penetration of an experimental resin system through the smear layer into the underlying dentin to form a hybrid layer that included both smear layer and underlying dentin matrix.	Increasingly higher concentrations of phenyl-P in 30% HEMA were used as dentin primers to improve the bonding of adhesive resins to smear-covered dentin.	The highest bond strength was registered using a concentration of 20% phenyl-P. For this formulation, TEM confirmed that dentin was demineralized through partial dissolution of crystals surrounding collagen.	A single-step primer containing phenyl-P at an optimal concentration is effective for self-etching and bonding.
Miyasaka & Nakabayashi (2001)^67^	To examine the effect of a phenyl-P/HEMA acetone-based primer on the tensile bond strength to moist dentin surfaces that were preconditioned with ethylenediaminetetraacetate (EDTA) to remove the smear layer.	Each dentin surface was primed with phenyl-P/30 wt% HEMA in acetone, with experimental primers being formulated at phenyl-P concentrations that ranged from 1 to 20 wt%.	Phenyl-P at 12 wt% achieved the highest tensile bond strength, significantly higher than 3, 5, 15, or 20 wt% phenyl-P concentrations.	The experimental phenyl-P/HEMA acetone primer is effective for bonding to EDTA-conditioned dentin.
Leal et al (2011)^57^	To evaluate the effect of the concentration of the acidic functional monomer on dentin bond strength and stability of a model two-step, self-etch adhesive.	Six self-etch primers were formulated using HEMA, GPDM, ethanol and water. Mass concentrations of GPDM were 0, 15, 30, 50, 70 or 100 wt%. Specimens were aged in distilled water (24 h, 6 mo, 1 y).	The bond strength after 6 months was similar to that at 24 h for 15 and 50 wt%, but significantly lower after 1 year. The 30 wt% concentration showed no difference in bond strength over the 1-year storage period.	A concentration of 50 wt% phosphate monomer seems to be a maximum for self-etch primers. A moderate concentration of 30 wt% showed a good balance between bond strength and stability.
Yazdi et al (2015)^113^	To investigate the effect of different concentrations of 10-MDP in one-step self-etch experimental adhesives on the micro-shear bond strength and degree of conversion.	Five experimental one-step self-etching adhesives containing 0, 5, 10, 15, and 20 wt% 10-MDP were used, with Clearfil S3 Bond (Kuraray Noritake) serving as control.	Higher bond strength was recorded for 10 and 15 wt% 10-MDP formulations in comparison to the other formulations, while the control adhesive and 10 wt% 10-MDP resulted in a significantly greater degree of conversion than that of the alternatives.	The amount of functional monomer in such adhesives influenced both the bonding performance and degree of conversion. 10 wt% 10-MDP appeared the most optimum concentration.
Amaral et al (2016)^3^	To investigate the influence of 4-META concentration and type of solvent on the degree of conversion (DC) and adhesive-dentin bond stability of experimental etch-and-rinse adhesives.	Experimental adhesives formulated with four different concentrations of 4-META (12, 20, 30, and 40 wt%) were tested.	The formulation containing 12 wt% 4-META presented with the lowest DC, while all other formulations showed statistically similar DC. All adhesives maintained the adhesive-dentin bond stability upon 6-month water storage, while only the adhesives containing 40 wt% 4-META maintained their bonding performance after 1 y.	Incorporation of high concentrations of 4-META improved the adhesive-dentin bond stability (1 y) of the experimental etch-and-rinse adhesives.
Kintopp et al (2020)^52^	To evaluate the influence of 10-MDP concentration and application mode of experimental adhesives on the micro-shear bond strength to dentin after storage in distilled water (24 h, 6 mo).	Five experimental adhesives were prepared with concentrations of 0, 3, 9, 12, and 15 wt% 10-MDP, which were applied to flat dentin surfaces in etch-and-rinse or self-etch bonding modes.	The 9 wt% adhesive formulation showed the highest bond strength values, while the 3 wt% adhesive formulation was most stable after storage. A strong negative correlation between 10-MDP concentration and pH was observed.	Formulations containing low concentrations of 10-MDP (up to 9 wt%) showed better results in terms of bond strength and bond stability over time.
Zirconia	Suh et al (2023)^100^	To investigate the shear bond strength and pH of a universal adhesive varying the concentration of 10-MDP.	Five experimental adhesives were prepared with 10-MDP ranging from 9 g to 12 g, which resulted in final compositions with 10-MDP ranging from 8.9 wt% to 10.5 wt% maximum.	There was a significant difference between all groups except between the two groups containing the highest concentration of 10-MDP (P>0.05). As the amount of 10-MDP increased, the shear bond strength initially increased and then decreased with the highest bond strength recorded for 9.7 wt%, whereas the pH decreased with increasing 10-MDP concentration.	This study found that increasing 10-MDP in the experimental adhesive lowered the pH and gradually improved shear bond strength, though bond strength declined once a concentration of 9.7 wt% was exceeded.
Yoshida et al (2006)^115^	To evaluate the shear bond strength of dual-cured resin luting cement to pure zirconium (99.9%) and industrially manufactured yttrium-oxide partially stabilized zirconia ceramic, and the effect of the 10-MDP primer and zirconate coupler (ZC) on bond strength.	Pure zirconium and zirconia ceramic specimens were left untreated or treated with experimental primers containing different concentrations of 10-MDP and ZC or a mixture of 10-MDP and ZC. 10-MDP was tested at concentrations of 0.1, 0.2, 0.5, 1.0, or 5.0 wt%.	The use of 10-MDP greatly increased bond strength to both substrates, with the results depending upon the concentration added, especially for bonding to pure zirconium.	The application of the mixture of 2 wt% 10-MDP primer and 1 wt% ZC was effective for bonding of dual-cured resin luting cement to zirconia ceramic.
Zirconia	Moraes et al (2012)^69^	To evaluate the influence of the acidic monomer type (phosphate acrylic monomer – PAM, or carboxylic acrylic monomer – CAM) and its concentration on the bonding performance and degree of conversion (DC) of single-solution model dental zirconia primers to yttria-stabilized polycrystalline dental zirconia ceramic.	The acidic monomers GPDM or mono-2-(methacryloyloxy) ethyl maleate (CAM) were added to a photocurable dimethacrylate at 10, 20, 40, or 60 wt%, containing 40 wt% ethanol.	Lower DC was observed for increased acidic monomer concentrations. The bond strengths of GPDM-based primers were generally higher than those of the CAM-based materials, irrespective of acidic monomer concentration or aging condition. After thermocycling, the bond strengths for PAM-based primers were still higher compared with those recorded for CAM-based primers.	Both the acidic monomer type and concentration had a significant impact on the adhesive performance of the primers. The primer containing 40 wt% GPDM showed the best overall bonding performance to dental zirconia.
Chen et al (2016)^17^	To examine the effects of PENTA as an alternative phosphate ester monomer for bonding of methacrylate-based resins to yttria-stabilized tetragonal zirconia polycrystals (Y-TZP) using a shear bond-strength method.	Experimental PENTA-containing primers (5, 10, 15, 20 or 30 wt% PENTA in acetone) were tested for their bond strength to Y-TZP. Bonding without the use of a PENTA-containing primer served as negative control, and a 10-MDP-containing primer was used as positive control.	Shear bond strengths were significantly higher for the 15 and 20 wt% PENTA formulations. ICP-MS, XPS and FTIR data showed that the phosphate content on the Y-TZP was concentration-dependent.	PENTA improves bonding to Y-TZP through chemical reaction with Y-TZP. Increasing the concentration of PENTA augments its binding affinity but not its bonding efficacy with zirconia.
Llerena-Icochea et al (2017)^60^	To evaluate the influence of adhesives with different 10-MDP concentrations on the shear bond strength of a resin cement to zirconia.	Six experimental adhesives were prepared with varying concentrations of 10-MDP (0, 3, 6, 9, 12, or 15 wt%). Three commercially available adhesives were evaluated: Single Bond Universal (3M Oral Care/Solventum), Adper Single Bond 2 (3M/Solventum), and Signum Zirconia Bond (Kulzer).	Significant differences were found between the commercial adhesives, while no differences were found between the experimental adhesives. A non-linear correlation was found between bond strength and 10-MDP percentage within the experimental adhesives.	The commercially available adhesives indicated for bonding to zirconia showed the highest bond strength.
Nagaoka et al (2017)^73^	To assess the chemical interaction and shear bond strength (SBS) between 10-MDP and zirconium oxide using magic angle spinning (MAS) nuclear magnetic resonance (NMR) and two-dimensional (heteronuclear correlation (HETCOR) NMR.	SBS tests were conducted to determine the effect of 10-MDP concentration on the bonding effectiveness to zirconia.	SBS tests revealed a 10-MDP concentration-dependent SBS with a minimum of 1 ppb 10-MDP needed.	Bond strength was found to be concentration dependent. The combination of both NMR techniques revealed not only ionic but also hydrogen bonding of 10-MDP to zirconia.
Chen et al (2017)^16^	To test the hypothesis that the concentration of 10-MDP in zirconia primers has no effect on the bonding efficacy through shear bond-strength testing of methacrylate resins to yttria-stabilized tetragonal zirconia (Y-TZP).	Five experimental primers containing 5 (5M), 10 (10M), 15 (15M), 20 (20M) or 30 (30M) wt% 10-MDP were tested to improve the composite-zirconia bond strength.	Shear bond strengths were significantly lower for group 5M when compared to groups 10M to 30M, which were not significantly different from one another.	10 wt% MDP appears to be the most optimal concentration for synthesizing zirconia primers for resin bonding.
Yoshida (2021)^114^	To investigate the effect of 10-MDP concentration in primers on the tensile bond strength, before and after artificial aging, of resin cements to zirconia.	Zirconia plates were bonded to stainless steel rods using commercial resin cements without functional monomers and were pretreated with one of the primers containing 0.5, 1.0, 2.0, 3.0, 4.0, or 5.0 wt% 10-MDP dissolved in ethanol, or one of 3 commercial ceramic primers containing 10-MDP and silane coupling agents. Untreated specimens were used as controls.	Bond strength in the non-pretreated group was significantly lower compared to that in the pretreated groups with 10-MDP-containing primers. Bond strength was concentration dependent, rising from 0.5 to 3.0 or 4.0 wt%. Pre-treated groups with the 4.0-wt% 10-MDP-containing primer performed better than the commercial ceramic primers.	Bond strength was dependent upon 10-MDP concentration. 10-MDP at 4.0 wt% seemed to outperform the commercial ceramic primers containing 10-MDP and silane.
De Paula et al (2021)^83^	To assess the effect of different concentrations of 10-MDP included in experimental ceramic primers on the degree of conversion (DC) and micro-shear bond strength (µSBS) of a dual-cure composite cement, and on the acidity neutralization potential of zirconia in comparison to HAp.	Experimental ceramic primers were formulated using 5, 10, 20, or 40 wt% 10-MDP as an acidic functional monomer and camphorquinone (CQ)/amine or 1-phenyl-1,2- propanedione (PPD) as photo-initiator system. Clearfil Ceramic Primer (Kuraray Noritake) was used as commercial control.	DC was not affected except when the concentration of 10 wt% 10-MDP in CQ primer and 5 wt% 10-MDP in PPD primer was reached, this compared to the control. The 5 wt% 10-MDP in CQ and PPD primers showed the highest µSBS compared with the positive control. Higher concentrations of 10-MDP induced significant DC and µSBS reduction.	The 10-MDP monomer ought to be kept at low concentrations in zirconia primers to avoid reduction of polymerization and bond strength of the composite cement.
Calamita et al (2023)^10^	To test the effect of different concentrations of 10-MDP and GPDM, separately or combined, on the micro-tensile bond strength to zirconia.	Experimental primers were made with 3, 5, or 8 wt% of GPDM or 10-MDP in absolute ethanol. Composite resin sticks were bonded to zirconia using the primers.	All experimental groups revealed similar bond strength compared to that of the positive control (Monobond N, Ivoclar), except for the primer containing 8 wt% GPDM, which resulted in lower bond strength.	Individually, each monomer is able to promote chemical binding to zirconia, although their combination does not improve performance. The most effective concentrations were 3 wt% 10-MDP and 5 wt% GPDM.
Titanium	Tsuchimoto et al (2006)^105^	To investigate the effect of a 4-MET- and 10-MDP-based primer on the tensile bond strength of two composite cements to titanium (Ti).	Ti plates were treated with six experimental primers consisting of, respectively, 10-MDP and 4-MET in concentrations of 0.1, 1, or 10 wt%, or were kept untreated (control).	The highest bond strength was registered in the 10 wt% 10-MDP group. Pre-treatment with each 10-MDP-based primer resulted in a higher tensile bond strength as compared to any 4-MET pre-treatment.	The data obtained strongly suggest that 10-MDP is effective to improve the adhesive performance of resin to titanium, this also in comparison to 4-META.
Self-adhesive materials	Shibuya et al (2019)^93^	To evaluate the influence of varying concentrations of 10-MDP on the micro-tensile bond strength, flexural strength, water sorption and solubility of self-adhesive composite cements.	Experimental composite cements were used containing three different concentrations of 10-MDP: 3.3, 6.6, or 9.9 wt%.	The bond strength of the 6.6 wt% 10-MDP cement formulation was significantly higher than that of the 3.3 wt%, while not for the 9.9 wt% formulation. Water sorption increased with 10-MDP concentration, while three-point bending and solubility showed no difference.	The authors suggest that 6.6 wt% 10-MDP seems an optimum concentration regarding the properties that were tested in the study.
Delgado et al (2021)^23^	To assess the influence of systematically varying concentrations of 10-MDP in a self-adhesive remineralizing and antibacterial composite, in comparison to a control containing 3% 4-META, on the polymerization kinetics and shrinkage, biaxial flexural strength (BFS) and Young’s modulus.	Self-adhesive composites were prepared using bulk, diluent monomers, 10-MDP (0, 5, 10, 15 and 20 wt%) or 4-META (3%) and CQ.	Rates of polymerization and degree of conversion increased linearly from 0-20 wt% 10-MDP, while shrinkage remained constant. Strength showed a peak at 5 wt% 10-MDP.	Peak strengths with 3 wt% 4-META or 5 wt% 10-MDP are hypothesized to be due to chemical bonding of monomers to the calcium-phosphate particles added for remineralization. Polymerization kinetics increased linearly with the concentration of 10-MDP that was added.


#### pKa

The application time of a self-etching primer containing a functional monomer is relevant, as longer times are known to improve etching efficacy and bond strength to dentin and even more to enamel. This is linked to the differences in (self-)etching efficacies found with different functional monomers.^120^ The pKa of the functional monomers is then extremely relevant to review their (self-)etching efficacy. Table 4 shows the pKa of some of the tested functional monomers as compared with orthophosphoric acid according to data found in the literature.^87^

**Table 4:** pKa of phosphoric acid and acidic monomers as found in the literature and calculated using ChemBio Draw Ultra (v.19.1)

Component	pKa
Orthophosphoric acid	2.0^1^
10-MDP	2.2^1^
4-MET	2.9^1^
MAC-10	4.7^2^
MMEP	3.1^2^
^1^ Taken from Salz et al (2005); ^2^ Calculated using ChemBio Draw Ultra (v.19.1) software.

The pKa is considerably important for the mineral-dissolution ability of the acidic monomer, which will also dictate whether it can effectively remove surface smear. The degree of smear-layer preservation directly affects bond strength.^42^ Other than lengthening the application time, bond strength is also highly dependent upon the concentration of the acidic monomer. This is usually technical information that is not released by the manufacturer. Increasing the content of acidic monomers in an adhesive formulation shows a positive correlation with a decrease in pH.^54^ Lower pH, on the other hand, can harm the stability of the adhesive ingredients in the product bottle, thereby decreasing the shelf life of the adhesive. Other clinical application modalities that are expected to improve bonding performance are actively rubbing the surface to intensify the local contact of the functional monomers with the surface and repeated application of fresh primer solution to the surface.

#### The peculiar case of inferior chemical adhesion to enamel

The mechanism and outcome of chemical adhesion onto enamel are fundamentally different from those to dentin, which can be explained by the differences in the structure and deposition of the mineral content. Functional monomers show a lower chemical reactivity to enamel HAp for several reasons. In enamel, HAp crystals are larger, the degree of crystallinity is higher, and there is a parallel crystal organization (as opposed to a crisscross orientation in dentin), making calcium ions harder to reach. ^65,119^ The observed variations also impact the deposition time of monomer-Ca salts.^119^ Nano-layering and/or stable Ca-salt formation of 10-MDP at the adhesive interface with enamel has been proven using X-ray diffraction (XRD) to be less intensive when compared to the interface formed at dentin.^126^

#### Interaction with hydroxyapatite (HAp)

As mentioned above, Yoshida et al (2001)^117^ introduced the adhesion-decalcification (AD) concept to elucidate the molecular interactions of acidic functional monomers, particularly those containing carboxylic or phosphate groups, with all HAp-rich mineralized tissues, although the example focused on dental hard tissues (Fig 2). According to this model, when acidic monomers contact HAp-rich substrates, their interaction occurs via a two-step mechanism. Initially, the acidic groups ionically bind to calcium ions at the HAp surface, concurrently removing phosphate (PO4^3-^) and hydroxyl (OH^-^) ions from the mineral structure.^89^ Subsequently, the behavior of these bonded acids is governed by the solubility of their calcium salts: acids forming salts with low solubility tend to remain bonded, thus minimizing demineralization and following the adhesion route, while those forming highly soluble salts detach from the surface, resulting in substantial decalcification and following the decalcification route.^117,132^

The solubility of the formed calcium-monomer complexes is relevant for bond longevity.^119^ The low dissolution rate of these salts is a critical determinant of the durability of the chemical bond, as it allows the adhesive interface to persist even under hydrolytic and mechanical challenge.^70^ Among these, 10-MDP has extensively been studied and is recognized for forming a highly stable calcium salt (Fig 4)^36,108,119^. Monomers such as GPDM, 4-MET, and PENTA follow the decalcification route and are generally unable to form stable calcium salts, compared to 10-MDP, as was reported in several studies using nuclear magnetic resonance (NMR), XRD, and FTIR spectroscopy techniques.^36,118,122^ Upon initial contact with HAp, 10-MDP rapidly forms a stable and water-insoluble 10-MDP_Ca salt directly at the tooth surface, establishing a strong chemical bond. If the interaction continues over time, still within the first 24 h, a secondary phase may occur in which dicalcium phosphate dihydrate (DCPD; CaHPO_4_·2H_2_O) precipitates on top of the existing 10-MDP_Ca phase.^129^ Although DCPD is more soluble in water than the 10-MDP_Ca complex, its formation in this context is not necessarily unfavorable. It may reflect a supersaturation environment and a continued chemical interaction with calcium and phosphate ions, indicating active mineral dynamics at the interface. Importantly, the underlying 10-MDP_Ca salt remains intact and anchored to HAp, preserving the stability and integrity of the adhesive interface.^84,129^ Thus, in contrast to interactions where DCPD is the primary product, such as in the case of GPDM at 24 h,^122^ its appearance here is secondary and does not compromise the durable bonding characteristic of 10-MDP. Since the other monomers producing unstable bonds follow the decalcification route, they have demonstrated higher etching ability, such as the GPDM-containing Optibond Universal (Kerr) was shown to be a strong etchant at enamel, extending deep within its subsurface.^31^

#### Interaction with dentinal collagen

For restorative polymeric networks to replace HAp in dentin, complete infiltration and thorough envelopment of the fully (E&R) or partially (SE) exposed collagen fibrils by resin are required. However, true enveloping, filling the nano-sized pores and tight junctions within the dentinal collagen network by mechanisms of penetration and adsorption, is extremely unlikely due to the nature and chemistry of the substrate as well as the monomers themselves.^6^ In fact, the interactions of monomers with the complex structure of collagen are difficult to study, and the behavior of monomers during the hybridization process in dentin remains largely unknown.^107^ Studies that employed techniques such as saturation transfer difference NMR have detected monomer-collagen interactions, such as 10-MDP/collagen complexation.^51^ The authors of the latter study argue that the hydrophobic moieties of the monomer complex with the hydrophobic collagen surface. 10-MDP was described to stably interact with collagen, as opposed to 4-META.^45^ Secondary chemical bonds, such as Van der Waals or hydrogen binding, and primary ionic bonds may play a role in monomer-collagen interactions, as pointed out by Vaidyanathan et al (2009) through the use of simulation models.^107^ FTIR analyses revealed new peaks corresponding to phosphate-related bonds (P=O, P–O–C), indicating formation of collagen-phosphate complexes for 10-MDP. Ultraviolet-Visible spectroscopy and docking studies showed that this interaction does not damage the triple-helix structure of collagen, showing that 10-MDP forms multiple hydrogen bonds with specific amino acids in collagen (eg, Hyp44, Gly16, Glu13).^50^

There are different factors that may make interactions with collagen either possible or difficult. For one, the steric hindrance of monomers in adhesive formulations directly affects their capacity to conform and attach to a binding site. Due to specific chemical structures, functional monomers experience different degrees of steric hindrance. Accordingly, Nurrohman et al (2015) reported lower steric hindrance for 10-MDP in comparison to GPDM or phenyl-P. This in turn favors adsorption of monomers onto collagen and subsequent phosphorylation, leading to guided biomineralization of collagen fibrils, an effect that depends upon the functional monomer used. Again, 10-MDP demonstrated denser extrafibrillar and intrafibrillar mineralization than other functional monomers.^76^ Moreover, previous computational studies also revealed that the spacer group plays a decisive role in interaction with collagen. Monomers with five or more spacer units increased their chances of conforming and binding to sites in dentinal collagen.^106^ It has been argued that polymerized resin could be bonded to collagen via -CONH bonds.^133^

Regarding bond longevity, stable calcium salts should be formed by functional monomers following the adhesion route in the AD model that describes the chemical interaction of acidic molecules with mineralized tissues.^132^ In particular, 10-MDP has been proven to contribute to collagen stability.^12^ In adhesive formulations containing HEMA, acidic monomers such as 4-META or 10-MDP can aggregate with HEMA, preventing them from being available to bond to HAp or collagen.

Specifically, with 10-MDP, it has been suggested that aggregates form with hydrophobic domains of 10-MDP oriented toward the center, surrounded externally by HEMA molecules, consequently restricting 10-MDP from participating in essential collagen interactions^46^. 10-MDP has also been shown to modify the dentin surface, mimicking the attachment of phosphorylated non-collagenous proteins to collagen, leading to the formation of phosphorylated collagen, in other words, allowing intrafibrillar mineralization of the collagen structure within hybrid layers.^92^

While water is essential for ionization of functional monomers, residual water in dentin, particularly unbound (free) water, is still a strong barrier to obtaining a durable adhesive-dentin bond. The amount of unbound water is greatly affected by the type/polarity of the contained water-miscible organic solvent in the dental adhesive and the bonding mode employed.^19,63^ A crucial factor in improving the interaction between functional monomers and dentinal collagen is controlling the environment in which this interaction takes place. One such method is the ethanol wet-bonding technique, which was introduced over 15 years ago to exchange interfacial water for ethanol, in the hope of being a better medium to facilitate resin to infiltrate the wet collagen network.^82^ The technique improves monomer-collagen interaction by replacing water with ethanol, thereby facilitating the infiltration of the adhesive into the collagen matrix. Ethanol plays a dual role here: it substitutes for unbound water within the collagen and helps eliminate water during air-drying phases, providing a suitable environment for ionization and chemical reaction with the dentinal mineral phase.^49^ Although ethanol wet-bonding theoretically enhances resin-dentin interactions, its widespread clinical application has been limited due to procedural complexity and inconsistency in results. Hence, this bonding protocol has not yet reached its hoped clinical acceptance since it only works for an E&R bonding mode, while adding an additional time-consuming step. Alternative solvents, such as isopropanol, as in Prime&Bond Active (Dentsply Sirona), have been explored to further refine this technique and improve its reliability. Others suggested a dimethyl sulfoxide (DMSO) pre-treatment strategy for durable bonding to dentin prior to the adhesive application.^2,98,99^

### Polymerization

Adding functional monomers to adhesives can affect polymerization efficiency, as has been addressed in some studies.^37,77^ Acidic monomers have been found to contribute to amine co-initiator inactivation via an acid-base reaction.^77^ This however depends on the type of photo-initiator and may involve complex molecular interactions. Hanabusa et al (2016) explored the functional monomers 4-META and 10-MDP, concluding that both interfered with polymerization initiated by camphorquinone (CQ)/amine-based systems.^37^ In fact, CQ used with amine co-initiators, such as ethyl-4-(dimethylamino)benzoate (EDMAB), resulted in lower polymerization-conversion degrees when compared to phosphine-oxide initiators. Diphenyl (2,4,6-trimethylbenzoyl) phosphine oxide (TPO) and bisacylphosphine oxide (BAPO) appeared less sensitive to polymerization inhibition by acidic monomers.^68^ Borate-based initiators and butanedione also appeared more efficient in the presence of acidic monomers than traditional CQ/amine polymerization-initiation systems.^4,77^ Some studies have also highlighted the use of acidic self-etch monomers to enhance dark-phase polymerization through amine-acid complexes, leveraging HAp-triggered self-cure mechanisms.^59^

Based on the chemical structure shown in Figure 3, most functional monomers are mono-methacrylates, possessing only one methacrylate group for co-polymerization, except for GPDM (2), Pyro-EMA (4), PENTA (5), and PEM-F (5). Mono-methacrylates cannot crosslink monomers and thus form only linear polymers. Monomers with longer spacer chains, such as 10-MDP, increase polymerization linearity even further.^138^ Studies evaluating the impact of increasing concentration of functional monomers on the polymerization properties and kinetics are scarce. Thanks to its high reactivity and flexibility, a higher 10-MDP concentration was found to enhance the degree of polymerization conversion.^13,23^ Reactivity is closely linked to the formation of hydrogen bonds, which occur when interactions take place between acidic groups and ions or molecules.^13^ If the monomers have a low steric hindrance, this will facilitate monomer-monomer conversion reactions by increasing the frequency of active site collisions. If 10-MDP is added to composite mixtures, it will rely on co-polymerization with dimethacrylate monomers to induce crosslinking. Interestingly, the degree of crosslinking increases with higher concentrations of 10-MDP.^23^ This effect is likely due to greater flexibility in the linear polymer chains, which results in shorter side chains compared to UDMA. This improved polymerization enhances both the strength of the cohesive bonding layer and the overall bond strength. Additionally, a higher degree of conversion leads to increased durability and stability of the adhesive interface.^121^

In other dental material formulations, such as ceramic primers and self-adhesive composites, functional monomers have been shown to influence polymerization. Studies demonstrate that using CQ alone, even at high concentrations in 10-MDP-based formulations, leads to an improved degree of conversion and crosslinking, validating this photo-initiator system as an effective stand-alone option for self-adhesive composite mixtures.^23^ In ceramic primers, the presence of 10-MDP in concentrations up to 5 wt% has been demonstrated to be optimal in reducing inactivation of CQ-amine due to acidic conditions, while maintaining good bond strengths.^83^

### Mechanical Properties

The addition of adhesive functional monomers, such as 10-MDP, has been reported to influence the mechanical properties of dental materials, though the available evidence remains limited. In self-adhesive cements, increasing the concentration of 10-MDP (from 3.3 to 6.6 and 9.9 wt%) enhanced bond strength but had no significant effect on three-point bending flexural strength.^93^ Zhou et al (2019) utilized micro-Raman spectroscopy and nano-indentation to investigate the nano-mechanical properties of adhesive interfaces containing 10-MDP, compared to control samples without the monomer. However, the formation of 10-MDP nano-layers was hindered by linear polymerization and interference from HEMA, leading to a reduction in quasi-static and storage moduli, which can compromise the mechanical properties.^138^ In later studies, an observed decrease in storage modulus at the adhesive-dentin interface in the presence of HEMA also supported earlier hypotheses suggesting that the hydroxyl group of HEMA may interfere with the nano-layering typically formed by 10-MDP.^101^ Nano-layering has been identified as a contributing factor in enhancing the strength of the adhesive interface. Yoshihara et al (2020) reported a higher Derjaguin–Muller–Toporov (DMT) modulus in interfaces containing 10-MDP nano-layered structures, which contributed to improved cohesive properties of the adhesive-resin layer.^123^

### Biocompatibility

Free monomers that can diffuse through dentin and eventually reach the pulp complex and its cell population pose a toxicity risk. Cytotoxicity studies that evaluated functional acidic monomers are scarce in the literature.^51^ The lipophilicity and functional groups of methacrylate monomers directly affect cell response.^27,32,131^ Studies on the relationship between molecular structure and cytotoxicity have demonstrated that the addition of a hydroxyl group to a monomer can increase its cytotoxic potential. Dipole moments, van der Waals volumes, topological distance between carbon and oxygen atoms in spacer chains, or the intrinsic states of atoms are chemical descriptors that play a role in cytotoxicity responses of dental monomers.^35^ Kurata et al (2011) argued that acryloyloxy-phosphoryl or acryloyloxy-carboxyl containing monomers were more cytotoxic compared to ether-carboxyl or amide-sulfo-containing monomers.^54^

Most studies have focused on evaluating the cytotoxicity and genotoxicity of dental adhesives containing different monomer blends. The cytotoxicity of individual monomers, such as 10-MDP and 4-META, has been tested in MTT assays.^51^ In fact, 10-MDP has been shown to affect and suppress odontoblast differentiation, even in low toxic concentrations, thereby disrupting mineralization. This impacts calcium interactions from odontoblast-like cells, potentially hindering mineralization and the formation of protective or reparative dentin. Kim et al (2015) identified the minimum toxic concentration of 10-MDP as 100 mM but found various inflammatory and cellular stress parameters altered even at lower concentrations. MTT assay revealed that 10-MDP affected cell proliferation in a concentration and time-dependent manner.^51^ 10-MDP showed significant toxicity to human dental pulp stem cells (hDPSCs) at 3 mM but had minimal effects on cytokine release. GPDM was less toxic, even at 4 mM, but increased Interleukin-1 beta (IL-1β) levels while reducing IL-6, IL-8, and IL-10. Despite signs of cytotoxicity, actual cell death for both monomers was reported to be minimal.^14^ Recent findings on the effects of 10-MDP_calcium salts on osteoblasts and fibroblasts revealed no cytotoxicity and no significant impact on apoptosis, mitochondrial membrane potential, or reactive oxygen species (ROS) levels.^139^ However, these salts inhibited the secretion of MMP2 and MMP9 in both cell types, which may be an interesting avenue for parallel research. Likewise, the findings of Ma et al (2025),^62^ suggest that 10-MDP_Ca salts exert several beneficial biological effects on dental pulp stem cells (DPSCs). These calcium salts promoted the migration and odontogenic differentiation of DPSCs without inducing apoptosis; they contributed to maintaining the cellular redox balance by reducing intracellular ROS and stabilizing the mitochondrial membrane potential. This was accompanied by the preservation of normal mitochondrial morphology, suggesting a protective role in mitochondrial integrity. Additionally, 10-MDP_Ca salts were again found to suppress the expression of MMPs in DPSCs.

The functional monomer 4-META, when compared to monomers such as HEMA, TEGDMA, and UDMA, has a 56-fold lower half-maximum inhibitory concentration (IC50) than UDMA, a 170-fold lower IC50 than Bis-GMA (0.03 mmol/L), and approximately twice as high IC50 as HEMA. 4-META has been incorporated into the resin matrix of experimental pulp-capping agents, in conjunction with novel hydrophilic acrylamide monomers, and has enhanced cell viability compared to commercial alternatives, such as Theracal LC (Bisco, Schaumburg, IL, USA).^130^

A recent systematic review on the cytotoxicity of dental adhesives, which belong to various adhesive strategies, does not mention the impact of the chemical composition of the material.^11^ This shows that the cytotoxic impact of various monomers used in commercial materials is still largely unknown and underexplored.

## RESULTS

### Do Formulations Containing Acidic Monomers Degrade Faster?

Degradation of monomers through hydrolysis is favored in acidic media. The acidic moieties in monomers and their characteristics significantly influence the degradation potential and shelf-life of adhesive formulations.^24^ Self-adhesive materials with lower pH tend to exhibit reduced shelf-life stability, highlighting the critical importance of chemical composition in ensuring material stability.^47,87^ Adhesive formulations without HEMA, utilizing methacrylamides and phosphonic acid-based monomers at higher pH levels, have shown enhanced stability over time.^20^ Monomers such as 4-META and 10-MDP have experimentally been verified to undergo ester cleavage, transforming into hydrolyzed by-products with little or no function,^64,75,102^ although it is important to mention that all resin monomers, whether acidic or not, are subject to esterase activity. In a laboratory study by Fujita et al (2011), it was found that unbound/unreacted 10-MDP did not necessarily impair long-term durability (tested after 20,000 thermocycles), as it did not decrease bond strength.^33^ In self-adhesive composites that were submitted to accelerated aging, the presence of acidic monomers did not seem to show a higher susceptibility toward degradation of the materials, long-term.^90^

### Is the Application Time of Adhesive Materials Containing Functional Monomers Relevant?

Several laboratory studies have varied the dwell/rubbing time of adhesives containing functional monomers, specifically, 10-MDP-containing adhesives, from a few seconds up to 2 min.^8, 39,86^ The effect has been measured when bonding to enamel and dentin. In short, yes, the application time is relevant, as has also been pointed out in meta-analytical data concerning universal adhesives.^38^ The consensus is remarkably consistent, with studies reporting that <10 s of active application results in inferior bond strength compared to longer application times.^8,86^ About 15–20 s of active rubbing is considered sufficient for the phosphate of 10-MDP to ion-exchange with HAp and build characteristic 10-MDP_Ca nano-layering; going longer rarely improves bond strength. Other studies evaluating monomers such as GPDM and 4-META, also found an application time of 20 s to be most efficient.^8,18^

### 10-MDP as the Most Reliable Acidic Functional Monomer

Since it was first introduced in the 1980s, 10-MDP has been extensively studied due to its superior bonding behavior and long-term performance compared to other functional monomers, as mentioned earlier. What sets 10-MDP apart is the specific molecular architecture that optimally spaces the reactive chemical groups and controls their interaction with both organic and inorganic components of the tooth (Fig 4). The molecular structure of 10-MDP features a long, linear alkyl spacer, a decyl (10-carbon) chain, linking the phosphate group to the methacrylate moiety.^129^ This structure confers several critical advantages: (a) a hydrophobic tail for interface stability: after ionic bonding to HAp and stable calcium-salt formation, the hydrophobic tail orients away from the tooth surface, reducing water sorption phenomena and potential hydrolytic degradation; this is crucial for preserving the bond in the moist oral environment over time; (b) spatial separation for functional group independence: the decyl spacer chain provides optimal spatial separation between the reactive phosphate group and the polymerizable methacrylate; this prevents steric hindrance, allowing each end of the molecule to interact effectively with its respective substrate, being HAp and resin monomers, without mutual interference; (c) self-assembly of 10-MDP molecules into nano-layers or ordered arrays, with the hydrophilic phosphate groups anchoring adjacent nano-layers through calcium bridging and the hydrophobic tails aligning inside the nano-layers, a uniquely highly organized structure formed at the adhesive interface. In particular, this may happen in water-rich zones near tubule orifices where sufficient Ca was released from dentin by the etching capacity of 10-MDP. Studies using transmission electron microscopy (TEM) and X-ray diffraction (XRD) have demonstrated the presence of these nano-layers, believed to result from stable ionic interactions between 10-MDP molecules and calcium ions (Fig 5).^119,125,126^ These layers have been proven to add strength to the adhesive interface, enhancing its mechanical properties and chemical stability.^123^

The amphiphilic characteristics of 10-MDP have been further verified when 10-MDP-based cleaners and primers are used as surface-decontaminating agents.^28,104^ When applied to dental surfaces contaminated by saliva, the hydrophobic segment of the 10-MDP molecule interacts selectively with organic saliva components, forming structured aggregates or micelle-like assemblies. These micelles encapsulate and isolate organic contaminants, significantly decreasing their affinity and adhesion to tooth surfaces. Consequently, the contaminants are detached and no longer interfere with bonding. In the form of a primer that is rubbed on the surface, even without rinsing it off, 10-MDP appeared very useful as a surface-decontamination agent prior to bonding, able to achieve unaltered bond strength.^28^ Additional studies should investigate the decontamination and bond-preserving effect of 10-MDP on blood-contaminated surfaces.

When 10-MDP is applied to dentin or enamel, the acidic phosphate group begins to interact with the mineral phase, initiating a mild demineralization process (Fig 2). This process removes some surface Ca^2+^, PO_4_^3–^, and hydroxyl (OH⁻) ions from the outermost HAp crystals in a selective etching process. Concurrently, the freed calcium ions (from the partial dissolution of HAp) interact with unreacted phosphate groups of 10-MDP, forming 10-MDP_Ca salts. A high adsorption of 10-MDP onto HAp has been verified under several environmental conditions.^7^ These salts have been found to be crystalline^129^ and low in solubility, allowing them to precipitate directly near the adhesive interface, hereby anchoring the adhesive in a chemical bonding mechanism. This salt formation is localized, it occurs at the surface and helps terminate further etching by passivating the interaction zone. Thus, the formed 10-MDP_Ca complexes stabilize the interface and limit continued demineralization.^119^ The etching phase comes first, before enough salt has accumulated to inhibit it. The phosphate groups initially compete with HAp for calcium; as they remove calcium from the mineral, they facilitate controlled dissolution. Only after a threshold concentration of calcium ions is reached, and the solubility limit of 10-MDP_Ca salt is exceeded, does precipitation occur, essentially sealing the interaction and protecting the remaining structure. Studies using XRD and TEM have confirmed the formation of nano-layered 10-MDP_Ca structures on HAp surfaces,^119^ further supporting the concept that etching and bonding occur in a coordinated, self-regulating manner. 10-MDP_Ca salt deposition had been hypothesized to co-occur with etching, a feature that is described to be distinctive for 10-MDP compared to the other functional monomers.^120^ The extent of interaction between 10-MDP and HAp is pH-dependent, with optimal salt formation occurring at acidic pH, where phosphate groups are protonated but still reactive.^129^

Even compared to monomers that are highly hydrophilic and have multi-functional acrylate groups, such as PENTA, enabling efficient penetration into collagen-rich areas, the durability of the bond after aging is notably stronger in the case of 10-MDP. Specifically, 10-MDP, especially in its low-soluble calcium salt form, considerably decreases HYP release, indicating enhanced resistance to collagen degradation by MMPs, as stated earlier.^36,50^ In composite resins containing calcium-phosphate particles, 10-MDP has been added for functionalization, impacting the mechanical properties and improving mechanical stability upon long-term water storage.^27,117^

For the best outcome in calcium-salt formation, 10-MDP should be combined in an ethanol mixture, with an optimal pH range of 2–7.^137^ The presence of dimers in 10-MDP preparations affects dental adhesive performance.^34,124^ Dimers possessing free phosphoric-acid groups can etch HAp surfaces but have limited polymerization potential due to steric hindrance, reducing bond strength. Certain dimers lack both etching and polymerization capability. Yoshihara et al (2015) demonstrated significant purity variations in commercial 10-MDP batches.^124^ Specifically, using ^31^P NMR spectroscopy, impurities such as dimers were detected, correlating with reduced enamel-etching efficacy and accelerated adhesive degradation. Pure 10-MDP maintained structural integrity and showed superior bonding durability over time. The dimers identified (through characteristic peaks in NMR spectra) differ significantly in stability and contribution to adhesive bonding compared to pure monomeric 10-MDP. Importantly, the formation of these dimers can negatively impact the bonding efficiency of the adhesive due to the decreased chemical reactivity, as has also been seen before.^125^ Thus, monitoring and adjusting 10-MDP purity, accounting for dimer presence, is critical for optimizing adhesive formulations, ensuring effective etching, and enhancing polymer quality. Further research is necessary to fully understand how these dimers influence adhesion and adhesive stability. Impurities or incomplete synthesis products within lower-grade 10-MDP formulations hinder the consistency and completeness of 10-MDP_Ca salt formation, thus adversely affecting adhesive longevity and enzyme-inhibition capabilities.

**Fig 6:**
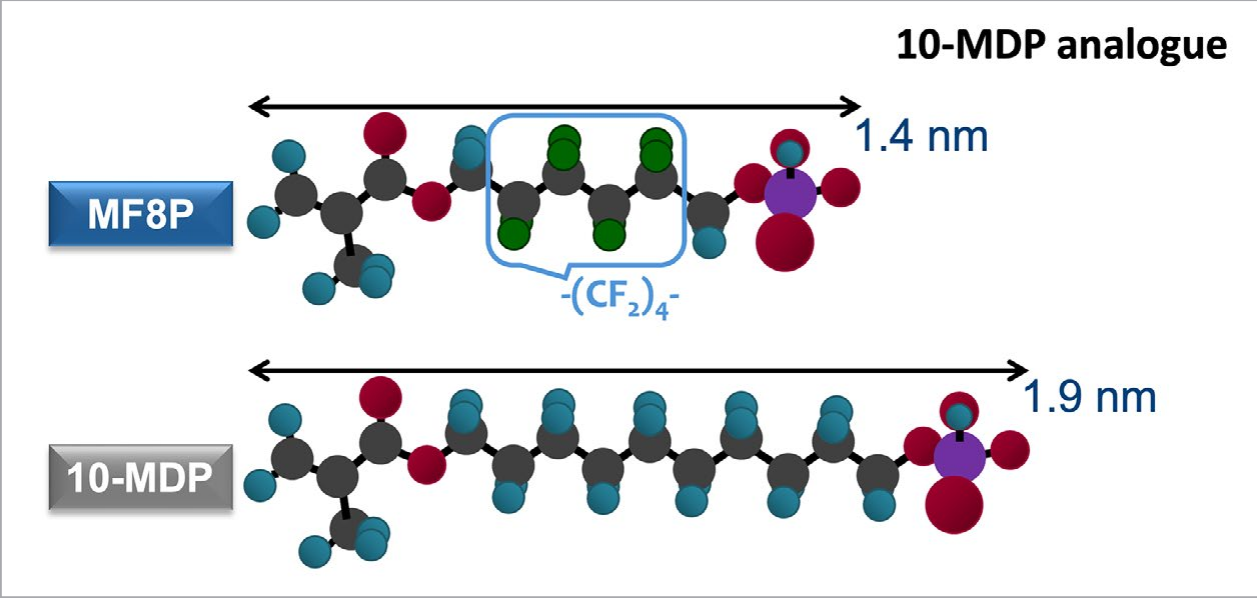
Molecular structure and size of the 10-MDP analogue functional monomer MF8P.

Recent research has explored alternatives to 10-MDP. Particularly, novel fluoro-carbon functional monomers such as 6-methacryloxy-2,2,3,3,4,4,5,5-octafluorohexyl dihydrogen phosphate (MF8P) (Fig 6). Techniques such as XRD and TEM have confirmed that MF8P readily interacts with Ca^2+^, forming stable MF8P_Ca salts. These salts also exhibit a pronounced ability to form nano-layered structures at the adhesive-dentin interface, closely mirroring the behavior of 10-MDP. These alternative calcium salts demonstrate the additional benefit of significantly higher bond strengths to dentin.^127^ This superior bonding capability is attributed to the robust chemical characteristics of fluorine-carbon bonds, known for their strong polarity and resistance to hydrolytic degradation. Although MF8P has a shorter molecular length compared to 10-MDP, its hydrophobic properties and resultant bond integrity highlight it as a promising candidate. Despite these promising findings, there are currently no commercial dental adhesives containing MF8P. This absence is likely due to the relatively high synthesis costs associated with fluorocarbon chemistry, which ultimately impact product pricing and industrial scaling feasibility.

## CONCLUSION

This review consolidates more than 30 years of evidence on acidic functional monomers used in contemporary dental adhesive technology. Three main insights emerge. First, 10-MDP remains the reference monomer for chemical bonding mechanisms, owing to its unmatched capacity to form hydrolytically stable Ca-salts, interact with dentinal collagen while self-assembling into reinforcing nano-layers, and also contribute to MMP-activity inhibition. It was found to be present in most self-etch/universal adhesives currently available in the market. Its effectiveness, however, depends on manufacturing purity; dimer or hydrolytic by-products undermine the etching efficacy and long-term stability, underscoring the need for rigorous quality control. Second, monomer architecture-spacer-chain length, hydrophobicity, and steric freedom governs the balance between etching aggressiveness, chemical bonding, polymerization kinetics, and consequently clinical longevity. This explains the inferior efficacy of shorter, more hydrophilic phosphate acrylates (e.g., GPDM) and carboxylates (eg, 4-MET), yet also rationalizes the superior performance of emerging fluorinated phosphates (MF8P, MF12P) that couple strong C-F bonds with effective Ca-bridging. Lastly, the bonding environment and formulation parameters (monomer concentration, solvent type, water content, initiator system, and co-monomers) modulate monomer reactivity, like molecular design. Strategies that tailor and control these parameters determine bond strength and interface stability.

Collectively, these findings provide a set of guidelines for structuring and engineering the next generation of durable self-adhesive materials.
